# Decoding Target Distance and Saccade Amplitude from Population Activity in the Macaque Lateral Intraparietal Area (LIP)

**DOI:** 10.3389/fnint.2016.00030

**Published:** 2016-08-31

**Authors:** Frank Bremmer, Andre Kaminiarz, Steffen Klingenhoefer, Jan Churan

**Affiliations:** Department of Neurophysics, Philipps-Universität MarburgMarburg, Germany

**Keywords:** saccade, smooth pursuit, monkey, area LIP, decoding

## Abstract

Primates perform saccadic eye movements in order to bring the image of an interesting target onto the fovea. Compared to stationary targets, saccades toward moving targets are computationally more demanding since the oculomotor system must use speed and direction information about the target as well as knowledge about its own processing latency to program an adequate, predictive saccade vector. In monkeys, different brain regions have been implicated in the control of voluntary saccades, among them the lateral intraparietal area (LIP). Here we asked, if activity in area LIP reflects the distance between fovea and saccade target, or the amplitude of an upcoming saccade, or both. We recorded single unit activity in area LIP of two macaque monkeys. First, we determined for each neuron its preferred saccade direction. Then, monkeys performed visually guided saccades along the preferred direction toward either stationary or moving targets in pseudo-randomized order. LIP population activity allowed to decode both, the distance between fovea and saccade target as well as the size of an upcoming saccade. Previous work has shown comparable results for saccade direction (Graf and Andersen, 2014a,b). Hence, LIP population activity allows to predict any two-dimensional saccade vector. Functional equivalents of macaque area LIP have been identified in humans. Accordingly, our results provide further support for the concept of activity from area LIP as neural basis for the control of an oculomotor brain-machine interface.

## Introduction

Primates perform saccadic eye movements in order to bring the image of an interesting target onto the fovea. In everyday life, it is often a moving target which attracts our interest. While computationally demanding, saccades to moving targets have been shown to be spatially accurate in monkey (Newsome et al., 1985; Keller and Johnsen, 1990) and man (Gellman and Carl, 1991; Kim et al., 1997, but see also Heywood and Churcher, 1981; Ron et al., 1989). It has been suggested that the oculomotor system uses speed and direction information about the visual target as well as knowledge about its own processing latency to program an adequate, predictive saccade. This hypothesis is supported by the finding that after lesions in the medio-temporal area (MT) of the macaque, which is known to be one of the key-areas for the processing of visual motion, the accuracy of saccades toward moving targets is reduced (Newsome et al., 1985; Schiller and Lee, 1994).

In primates, several cortical and subcortical brain regions have been implicated in the control of saccades (for review see e.g., Gaymard et al., 1998; Munoz, 2002). One of these regions is the lateral intraparietal area (area LIP) in the intraparietal sulcus. Many neurons in area LIP are active prior to saccades (Barash et al., 1991a) and have spatially matching visual and saccadic motor fields (Barash et al., 1991b; Platt and Glimcher, 1998). It is unclear, however, how LIP neurons behave when the distance between fovea and current target position on the one hand and appropriate saccade amplitude on the other hand are not identical as is the case for saccades toward moving targets. Given that neurons in area LIP receive strong input from area MT (Blatt et al., 1990), we considered it likely that area LIP is also involved in the programming of saccades toward moving targets. We recorded from single neurons in area LIP of the rhesus macaque while animals performed amplitude-matched saccades to stationary and moving targets. In about 40% of the neurons we found differential activation for the two types of saccades. At the population level, however, this effect was balanced out. By employing a combined Bayesian classifier and maximum likelihood approach we show that presaccadic LIP population activity allows to decode both, the distance between the fovea and a visual target as well as the amplitude of an upcoming saccade. Functional equivalents of macaque area LIP have been identified in human parietal cortex. Together with previous results on the decoding of saccade direction from population activity in area LIP (Graf and Andersen, 2014a,b), our results provide further evidence for the concept of an oculomotor based human brain-machine interface.

## Materials and methods

Extracellular recordings were performed in 2 hemispheres of two male macaque monkeys (Macaca mulatta; monkey K: 10.4 kg and monkey C: 9.5 kg). All procedures were in accordance with guidelines on the use of animals in research (European Communities Council Directive 86/609ECC).

### Animal preparation and experimental equipment

Before surgeries, animals were premedicated with atropine. Initial anesthesia was performed using Ketamine (monkey C) or Rompun/Ketamine (monkey K) respectively. Subsequently anesthesia was maintained by i.v. injection of either pentobarbital sodium (Nembutal; monkey C) or propofol (monkey K). Additionally, local analgesics were administered as needed.

After initial training, monkeys were implanted with a head holding device and (monkey C only) two scleral search coils (Judge et al., 1980). After final training, a recording chamber was implanted above the intraparietal sulcus in a second surgery. In monkey C, a stainless steel chamber was placed at P 3 L 15 at an angle of 45° relative to the vertical on the basis of a presurgical MRI scan. Both, chamber and head holding device were fixed with self-tapping screws and covered in dental acrylic (Technovit 4004). In monkey K, the chamber was placed at the same coordinates. The chambers were fixed with titanium screws only. Analgesics and antibiotics were applied postoperatively in both monkeys. Recordings started after full recovery of the animals (at least 1 week after surgery).

During training and experiments the monkeys were seated in a primate chair with their head fixed. Eye position was measured using the scleral search coil technique (monkey C) or with an infrared eye-tracking system (ET50, Thomas Recording, Gießen, Germany) (monkey K). In both cases eye-position was sampled at 250 Hz. Animals were rewarded with liquid (water or apple juice) for each correct trial. Data acquisition and visual stimulation was controlled using PC-based in-house software (NABEDA). For monkey K all stimuli were generated using the PC-based software *Neurostim* and presented on a CRT display covering the central 26° × 20° of the visual field, which was placed 89 cm in front of the animal. For monkey C stimuli were generated using a mirror galvanometer back-projecting targets (red dot diameter: 0.8°, luminance: 0.4 cd/m^2^) on a translucent screen placed 0.48 m in front of the monkey. Here, the screen subtended the central 90° × 90° of the monkey's visual field.

### Eye movement paradigms

**Figure 1:**
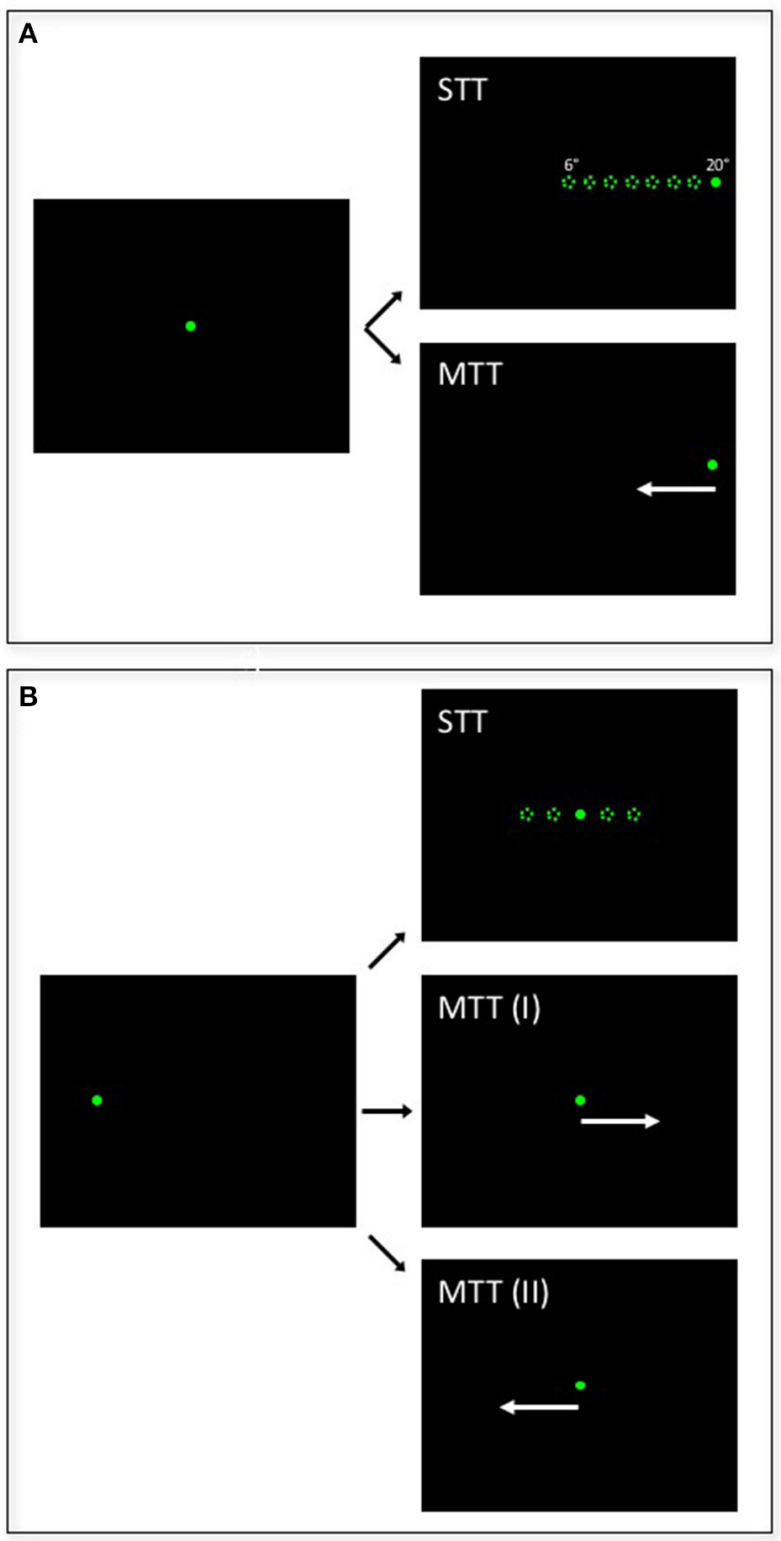
**Illustration of the saccade paradigms**. Each trial started with monkeys fixating a central (**A**: monkey C) or peripheral (**B**: monkey K) target for 1000 ms. Then, the target jumped in the preferred saccade direction of the cell under study to one of eight (**A**: monkey C) or five (**B**: monkey K) different distances and remained stationary for another 1000 ms in stationary-target trials (STTs). In moving-target trials (MTTs), the target jumped either to the most eccentric position and immediately started to move centripetally (**A**: monkey C) or it jumped to the central target position (**B**: monkey K) and started to move either in the same direction, thereby inducing forward pursuit (MTT-I), or in the opposite direction, thereby inducing backward pursuit (MTT-II).

All experiments were performed in complete darkness. For each cell we first determined the preferred saccade direction (PSD). Each trial started with fixation of a central target [(x, y) = (0°, 0°)] for 1000 ms. Then, the fixation target was switched off and a saccade target appeared at one of four possible locations on the cardinal axes (left, right, up, down) at 10° degrees from the fovea [i.e., (x, y) = (+10°, 0°), (−10°, 0°), (0°, +10°), or (0°, −10°)]. Monkeys were required to make a saccade and keep fixation until the end of the trial (2000 ms). The PSD was defined as the saccade direction associated with the largest perisaccadic response as determined from a 300 ms wide response window, centered on saccade onset (±150 ms). In case of similar saccade related discharges for two cardinal directions, we defined the angle bisector as the PSD. In all subsequent recordings, saccades to either stationary or moving targets were always in the PSD of the neuron under study. Each trial started with the fixation of either a central (monkey C, Figure 1A) or a peripheral target (monkey K: 6.4° eccentricity, Figure 1B). In the *stationary-target trials (STTs)*, the target jumped after 1000 ms to one of five (monkey K) or one of eight (monkey C) different positions and remained stationary. In monkey C, saccade target distances ranged from 6° to 20° eccentricity in two-degree steps (6°, 8°, 10°, 12°, 14°, 16°, 18°, 20°). In monkey K, due to the spatial limitation of the visual display, saccade target distances ranged from 7° to 12° (for vertical saccades), or 9° to 14° (for horizontal saccades), in equidistant steps. In the *moving-target trials (MTTs)*, the target either jumped to the most peripheral position (20°) and immediately thereafter started to move centripetally (monkey C) or it jumped toward the middle position of the stationary targets and immediately thereafter started to move either in the same direction as the saccade (inducing forward pursuit, MTT-I) or in the direction opposite to saccade direction (inducing backward pursuit, MTT-II). In all cases, the pursuit target moved for 1000 ms at a certain speed (monkey C: *v* = 30°/s; monkey K: *v* = 6.4°/s). Animals received liquid rewards for correctly performing the eye-movement tasks.

### Data analysis

Data were analyzed using Matlab 2015b (The Math Works Inc., Natick, USA). Saccades were detected using a velocity criterion (40°/s). Saccade onset was defined as the point in time when the eye velocity exceeded this criterion for three consecutive samples (12 ms). All data were aligned to saccade onset. As a first step, we determined if neurons were tuned for saccade direction. To this end, we analyzed perisaccadic activity for saccades into four different directions (left, right, up, and down) within a fixed perisaccadic window, i.e., from 150 ms before until 150 ms after saccade onset. These four values were compared with baseline activity, i.e., spontaneous activity from 650 to 250 ms before saccade onset, using an ANOVA on ranks. By choosing this latter value (i.e., 250 ms) we avoided interference of baseline discharges with visually induced and/or presaccadic burst activity. The analysis allowed us to determine the preferred saccade direction.

For those neurons, which were tested for saccades toward stationary and moving targets, we determined, whether their presaccadic activity was tuned for target distance or saccade amplitude by means of an ANOVA on ranks. This analysis was based on neural discharges within a brief, presaccadic time window Δt = −80 to 0 ms, i.e., from 80 ms before saccade onset until saccade onset.

In a final step, we determined whether the activity for saccades toward stationary and toward moving targets was significantly different. To this end, we compared the mean activity within the same time window (−80–0 ms) for saccades toward moving targets and amplitude-matched saccades toward stationary targets by means of a Mann-Whitney rank test. In all cases (i.e., ANOVAs and Mann-Whitney test), a *p*-value of *p* < 0.05 was considered as threshold for statistical significance.

### Decoding of target distance and saccade amplitude

The major goal of our study was to determine whether activity from a population of LIP neurons is suited to decode the distance of a saccade target, or the amplitude of an upcoming saccade, or both. A number of recent studies have investigated related issues, among them Sereno and Lehky (2011) and two studies by Sajad et al. (2015, 2016). Sereno and Lehky have shown that population activity from area LIP allows to decode visual information at high spatial resolution. This result was surprising given the relatively large visual RFs of LIP neurons. Sajad and colleagues investigated the transition from sensory to oculomotor signals. They analyzed neural activity from the frontal eye-fields (FEF) with respect to different reference frames (head-, gaze-, and target-centered) in a head-unrestrained monkey. Different from their approach, however, we employed a Bayesian Classification together with a maximum likelihood approach. As is rather typical for experiments with non-human primates, neurons were not recorded simultaneously. Accordingly, in our analysis a set of trials refers to a synthetic data set in which a number of experimental trials were drawn at random without replacement from all available trials recorded from a neuron. This is a common and useful way to simulate population codes in the brain from single neuron data (Salinas and Abbott, 1994). It should be noted, however, that this approach ignores potential effects of noise correlations on neural representation. Correlations can enhance, degrade, or have no effect on the information that can be extracted from a population code (Averbeck et al., 2006).

It has been shown before that activity from a population of neurons from area LIP can be used to predict the direction of an intended saccade (Graf and Andersen, 2014a,b). In a sense, our study can be considered a complement of these two previous studies. Graf and Andersen aimed to classify decoded direction into eight bins, basically reflecting the eight different imposed saccade directions. In our study, we have presented targets at eight (monkey C) or five (monkey K) different distances. The spacing of the saccade targets resulted in a quasi-continuum of saccade amplitudes. Hence, we had to decide whether to decode saccade amplitudes as continuous variable or classified into bins. In order to complement the studies by Graf and Andersen, we decided to classify saccades into eight or five amplitude bins, respectively. Different from these two previous studies, however, we did not employ a memory guided but a visually guided saccade task. We decided for this kind of task because it comes closest to situations in everyday life, where an object of interest enters the visual field and triggers an immediate saccade. Nevertheless, our goal was to determine, whether activity from area LIP could be used to predict the amplitude of an upcoming saccade. Such a *predictive* decoding, i.e., decoding from activity *before* an eye-movement, would be required for the control of a brain-machine interface. Hence, our further analyses had to be based on a brief time window prior to saccade onset, containing most likely visual *and* motor signals. It has been shown, that for the programming of saccades, information about target position is only used up to about 80 ms before the onset of a saccade (Becker and Jürgens, 1979). Accordingly, we used this very time window, i.e., from 80 ms before saccade onset until saccade onset, to apply our decoding technique.

As a first step we determined the spike-count distributions for each neuron in the time window Δt = −80 to 0 ms prior to saccade onset. In general, these distributions can be estimated using parametric or non-parametric approaches (Graf et al., 2011). A parametric approach assumes a certain activity distribution profile (e.g., Poisson) and the task would be to estimate the parameters of the probability model that best account for the observed data. The non-parametric approach, in contrast, uses the observed spike-count distributions as an empirical estimate of the underlying probability function and whereas does not assume any particular probability model. Since we had the relevant data at hand, we decided for the non-parametric approach.

The observed distribution for each neuron provides a critical quantitative link (in statistical terms, a “likelihood function”) between the parameter under study and the neuron's response. In our case, we aimed to determine if LIP activity prior to a saccade contains the relevant information to decode (i) target distance, or (ii) the amplitude of the upcoming saccade, or both. Accordingly, for the five (monkey K) or eight (monkey C) target distances, we computed the spike count distributions in 5 Spikes-per-second-bins, i.e., we classified activity levels in bins, with each bin covering an activity range of 5 Spikes-per-second: 0–5 Spikes/s, 5–10 Spikes/s, 10–15 Spikes/s, etc. The same procedure of binning neural activity was applied with respect to saccade amplitudes. Given the quasi continuous range of observed saccade amplitudes (see below), we classified the observed saccades into five (monkey K) or eight (monkey C) bins. Bin size was chosen to achieve an approximately equal number of saccades within each bin. This dissociation of relating neural activity either with target distance or saccade amplitude was important because even for stationary targets, saccade endpoints show a certain degree of variation, i.e., presentation of the same saccade target does not always result in the same saccade amplitude (van Beers, 2007; Ohl et al., 2011). This effect is even stronger in case of a moving target. Here, the distance of the initial appearance of a moving target varies systematically from the location (i.e., amplitude) at which the eye reaches the target by an initial saccade. By computing the two spike count distributions for the target distances as well as for the saccade amplitudes we determined for each neuron the probability “p” of a neural response “R” given a certain target distance “xT” and/or a certain amplitude of an upcoming saccade “xS”:

- Target distance: *p*(*R*|*xT*)
- Saccade amplitude: *p*(*R*|*xS*)

Yet, decoding requires an additional computational step: it implements the reverse direction of inference. Accordingly, our decoding resulted in an estimate of the probability of (i) a certain target distance p(xT) and (ii) of a certain saccade amplitude p(xS), given an observed response:

- Target distance: *p*(*xT*|*R*)
- Saccade amplitude: *p*(*xS*|*R*)

These two conditional probabilities are related via Bayes' rule:

(1a) $$\begin{array}{r} \text{Target distance}:\;p\left(xT|R\right)\;=\;\frac{p\left(R|xT\right)*p\left(xT\right)}{p\left(R\right)} \end{array}$$

(1b) $$\begin{array}{r} \text{Saccade amplitude}:\;p\left(xS|R\right)\;=\;\frac{p\left(R|xS\right)*p\left(xS\right)}{p\left(R\right)} \end{array}$$

Here, *p*(*xT*) indicates the probability of the occurrence of a given saccade target distance. For each animal, these probabilities were equal, i.e., 1/8 for monkey C and 1/5 for monkey K. In both cases, *p*(*R*) indicates the overall, i.e., unconditioned probability to find a certain activity value as determined from the overall saccade related discharges in bins of 5 Spike/s. Accordingly, *p*(*xT*|*R*) is the posterior probability of all possible target distances given the neural evidence, and *p*(*xS*|*RR*) is the posterior probability of all observed saccade amplitudes, again, given the respective neural evidence. Following this approach, the decoders constructed for each neuron a probabilistic look-up table which was used to transform an observed spike-count into an expression of the relative likelihood of all possible (i) target distances or (ii) saccade amplitudes. To minimize the influence of neural noise, we presumed that the brain relies on a population code for target distance as well as for saccade amplitude. Assuming statistical independence among N neurons, the optimal way to combine probability distributions across a population is to take their product, or, preferably, the sum of their logarithms:

(2a) $$\begin{array}{r} \text{log p }\left(xT|R_{population}\right)\;=\;\sum_{i=1}^{N}\log p\left(xT|R_{i}\right) \end{array}$$

(2b) $$\begin{array}{r} \text{log p }\left(xS|R_{population}\right)\;=\;\sum_{i=1}^{N}\log p\left(xS|R_{i}\right) \end{array}$$

In a final step, the respective bins associated with the maximum a posteriori log-likelihood (i.e., the MAP estimate), i.e., log p (*xT*|*R*_*population*_) or log *p* (*xS*|*R*_*population*_) were considered (i) decoded target distance or (ii) decoded saccade amplitude.

We employed cross-validation to ensure that the results of the population-decoding reflected reliable characteristics of the neural code for target position as well as saccade amplitude. In all cases, i.e., for determining the target distance as well as the amplitude of an upcoming saccade in *STTs*, we drew randomly without replacement approximately 80% of the available trials for each neuron (“training-set”). Decoding for the STTs was then performed on the remaining 20% (“test-set”) of trials for each neuron. This procedure was repeated a 100 times for each neuron. Importantly, decoding for the MMTs was performed with all data from MTTs, but it was based on the decoder built from the STTs. As a further control, we assigned the observed spike count distributions to randomly chosen (without replacement) target distances or saccade amplitudes. If the correct performance of the decoder would have relied on the correct assignment of spike count distributions to target distances or saccade amplitudes, this procedure should result in random decoding performance.

## Results

### Saccade related discharges

We recorded from 131 neurons in area LIP of two macaque monkeys: 99 from monkey K and 32 from monkey C. In a first step, we tested neurons for a saccade related response in a visually guided saccade task. Monkeys first had to fixate a central target which was presented for 1000 ms. Then the fixation target was switched off and a saccade target appeared at one of four possible locations on the cardinal axes (right, left, up, or down) at 6.4° (monkey K) or 10° (monkey C) eccentricity. 80/131 (61%) of the neurons revealed a significant perisaccadic discharge as compared to baseline (ANOVA on ranks, 4 df, *p* < 0.05), 55/99 (56%) from monkey K and 25/32 (78%) from monkey C. A visually guided saccade paradigm does not allow to dissociate visually induced and saccade-related discharges. We presume that the activity as reported in this paper carries both types of information. We could have aimed to dissociate visual from motor-related components by employing an overlap or a memory-guided saccade paradigm (Barash et al., 1991a,b; Colby et al., 1996). Yet, there were three major reasons for staying with the visually guided saccade paradigm. Firstly, it comes closest to everyday like scenarios, where an object catches our immediate interest by triggering a quasi-reflexive saccade. Secondly, overlap or memory intervals, which separate a visual event from a motor event (saccade), allow for further ongoing neural signal processing. This processing, however, would not have been under our experimental control and might have varied in its temporal and spatial aspects across trials. Thirdly, and most importantly, the central goal of our study was to test, whether the activity from a population of LIP neurons multiplexes information and carries simultaneously signals concerning the distance of a visual target as well as the amplitude of an upcoming saccade. Accordingly, in the following, while we chose the term *saccade-related or perisaccadic activity* as measured in our study, we acknowledge that part of this activity was most likely visually driven.

### Tuning for target distance and saccade amplitude

**Figure 2:**
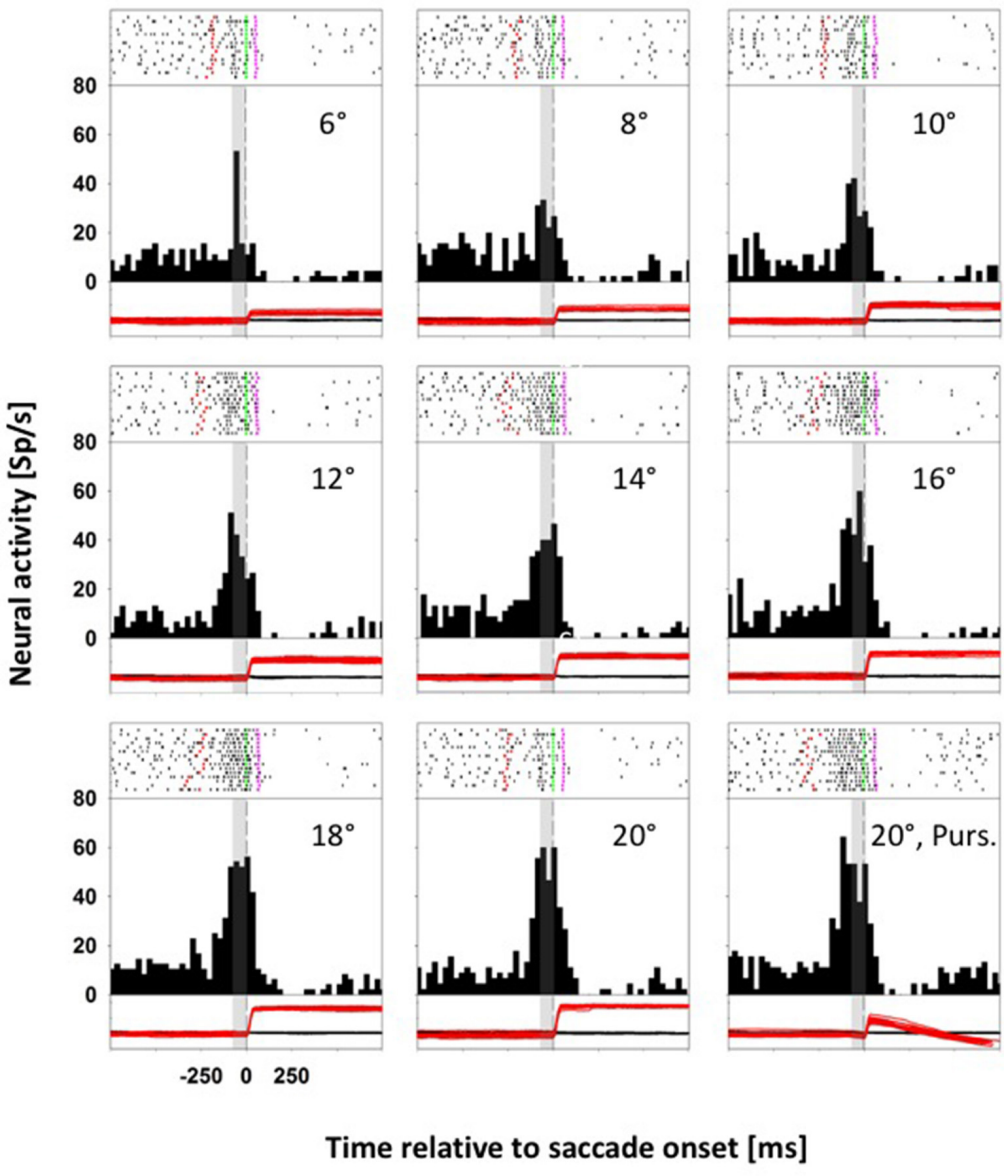
**Data from an LIP neuron exhibiting differential activity for saccades toward stationary and moving targets**. In each panel, spike rasters for individual trials are shown at the top, response histograms are shown in the middle and eye position traces are shown at the bottom (black curve: horizontal eye position; red curve: vertical eye position). All data are aligned to saccade onset. In the spike rasters, the green lines indicate saccade onset, and the pink lines saccade offset. The dashed vertical lines in the histograms indicate saccade onset. The gray rectangles indicate the temporal window employed for analyzing perisaccadic activity (from −80 to 0 ms relative to saccade onset). For this neuron, perisaccadic activity in moving-target trials (lower right panel) was significantly higher than activity in amplitude matched stationary-target trials (Mann-Whitney rank test, *p* < 0.05).

Neurons with a direction specific, saccade related response were tested for their responses for saccades toward stationary and moving targets. For monkey C, we presented eight stationary targets and one moving target in pseudorandomized order: for monkey K, we presented five stationary targets and two moving targets (for details see Methods). Figure 2 shows data from a representative neuron from monkey C with differential activity for saccades to eight stationary targets and one moving target. The preferred saccade direction of this neuron was upward. Accordingly, across trials, stationary targets were presented in pseudorandomized order in two-degree steps between 6° and 20° eccentricity above the fovea on the vertical meridian (VM), eliciting saccades covering a quasi-continuous amplitude range between 4.5° and 20.2°. In this recording, saccade latencies ranged from 136 to 348 ms, with an average latency of 231 ms. The *moving-target trials* (*MTTs*. Lower right panel) were presented interleaved with the *STTs*. For this neuron, given its direction preference for upward saccades, the moving target appeared (after central fixation) at 20° in the upper visual field on the VM and immediately started to move centripetally (downward) at 30°/s. In this condition, saccade latencies ranged from 240 to 348 ms, with an average latency of 304 ms. Accordingly, the animal had to adjust the amplitudes of its initial saccades to reach the moving target properly. Amplitudes of these initial saccades ranged from 5.5° to 11.9°, with an average amplitude of 9.6°. Spike rasters for individual trials are shown at the top of each panel, response histograms are shown in the middle and eye position traces (black: horizontal; red: vertical) are shown at the bottom. All data in each panel are aligned to saccade onset. The gray rectangle in each histogram and eye-movement panel indicates the temporal window used for the comparison of the saccade related activity in the *STTs* and the *MTTs* (−80 to 0 ms relative to saccade onset). This neuron responded significantly for upward saccades (ANOVA on ranks, 8 df, *p* < 0.0001) and it was significantly tuned for saccade amplitude in the STTs (ANOVA on ranks, 7 df, *p* < 0.0001). For *STTs*, the neuronal activity increased with increasing saccade amplitude, saturating for saccade amplitudes of 16° or more. For statistical comparison of saccade related activity in *STTs* and *MTTs*, we considered only those saccades from *STTs* which covered the same amplitude range as the saccades from the MTTs. This neuron revealed significantly stronger activity in *MTTs* as compared to STTs of the same amplitude (Mann-Whitney rank test, *p* < 0.05).

**Figure 3:**
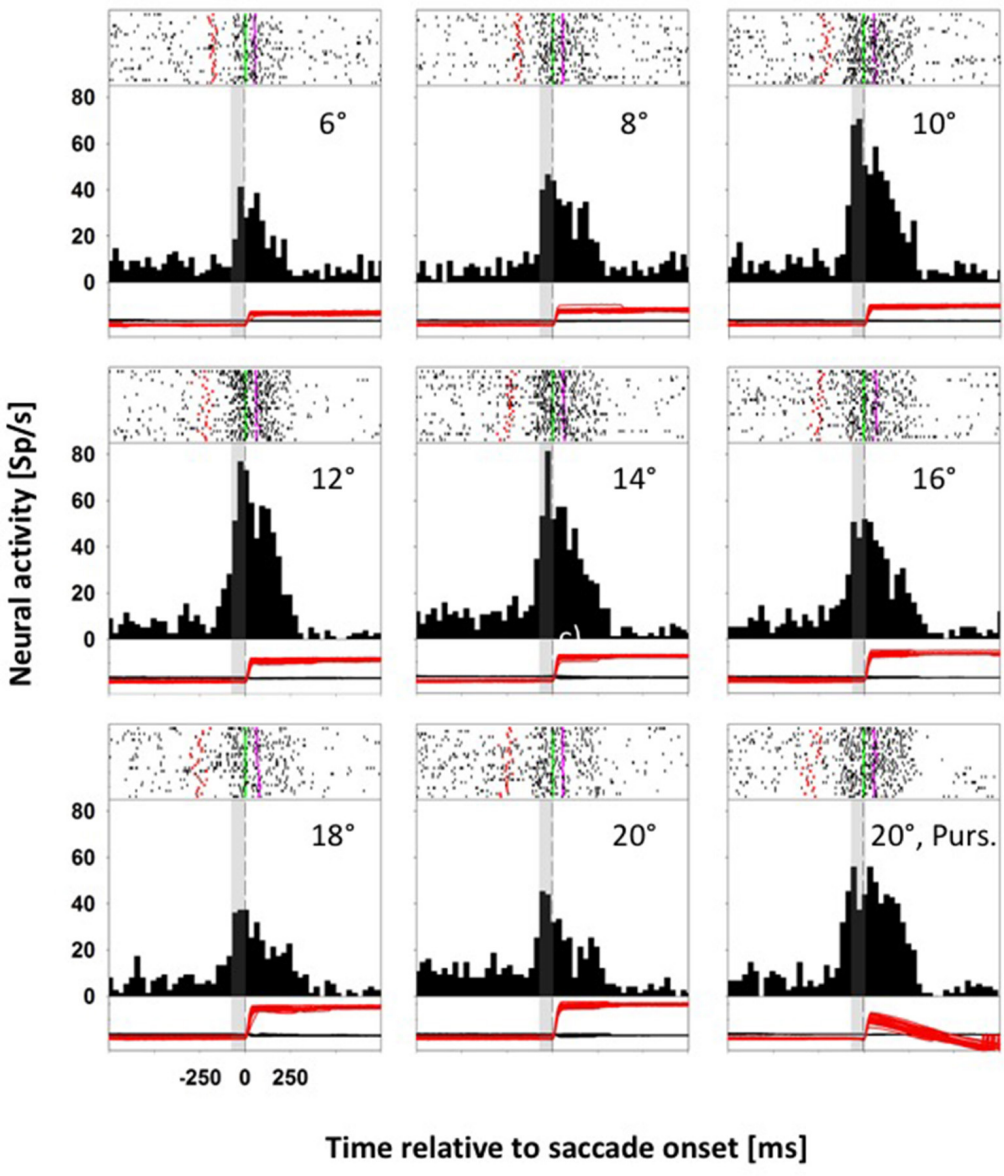
**Data from an LIP neuron exhibiting similar activity for saccades toward stationary and moving targets**. Conventions as in Figure 2. For this neuron, perisaccadic activity in moving-target trials (lower right panel) was not significantly different from activity in amplitude matched stationary-target trials (Mann-Whitney rank test, *p* > 0.05).

Figure 3 shows data from a second neuron, also from monkey C, which responded significantly for upward saccades (ANOVA on ranks, 8 df, *p* < 0.0001). Again, we quantified the perisaccadic discharges for eye movements toward the eight different stationary targets. Like before, the activity of this neuron was tuned for saccades toward stationary targets (ANOVA on ranks, 7 df, *p* < 0.0001). But here, activity values for saccades toward moving and for amplitude-matched saccades toward stationary targets were not significantly different (*p* > 0.05, Mann-Whitney rank test).

For our analyses we employed a brief, presaccadic interval from −80 to 0 ms with respect to saccade onset. The purpose for choosing this time window was two-fold. First, it was early enough to contain visually induced activity and late enough to contain saccade-related motor signals. Second, it was presaccadic. Hence, if our decoder for saccade amplitude would correctly perform (see below), this would mean that we would be able to predict the amplitude of an upcoming saccade. Considering this very brief presaccadic analysis window and the amplitude ranges tested in our paradigms, 33/80 (41%) of the neurons were tuned for the amplitude of the upcoming saccade toward a stationary target: 15/55 (27%) neurons from monkey K and 18/25 (72%) neurons from monkey C.

The proportion of tuned cells varied strongly between the two animals, which simply might have been due to the small amplitude ranges employed for monkey K as compared to a much wider range employed for monkey C (see Methods for details). We aimed to verify this hypothesis and first determined the distribution of absolute amplitudes of saccades toward stationary targets for both animals. For monkey K, 5% of the saccades were smaller than 5.2° and another 5% were larger than 12.5°. For monkey C, these values were 5.8° and 19.0°. In order to make saccade tuning data comparable across the two animals, we analyzed the tuning for the smaller saccade range (5.2° to 12.5°) in monkey C. As expected, only 36% of the neurons were significantly tuned. This value (36%) comes close to the value measured in monkey K (27%). Accordingly, we conclude, that the differences in proportion (27 vs. 72%) were mainly due to the different amplitude ranges that were tested in the two animals.

14/33 (42%) of these amplitude-tuned neurons exhibited differential activation for amplitude matched saccades in *STTs* and MMTs: 5/15 (33%) from monkey K and 9/18 (50%) from monkey C. Remarkably, considering these latter neurons, there was no systematic difference in activity at the population level between STTs and MTTs. For both monkeys, the number of neurons for which activity was larger in MTTs as compared to amplitude-matched STTs was about the same as the number of neurons for which the opposite was true. Signed rank tests confirmed this rather qualitative analysis: at the population level, activity levels for the two saccade types were not significantly different from each other: *p* > 0.4 for STT vs. MTT data from monkey K, *p* > 0.6 for data from monkey C.

### Eye movement behavior

**Figure 4:**
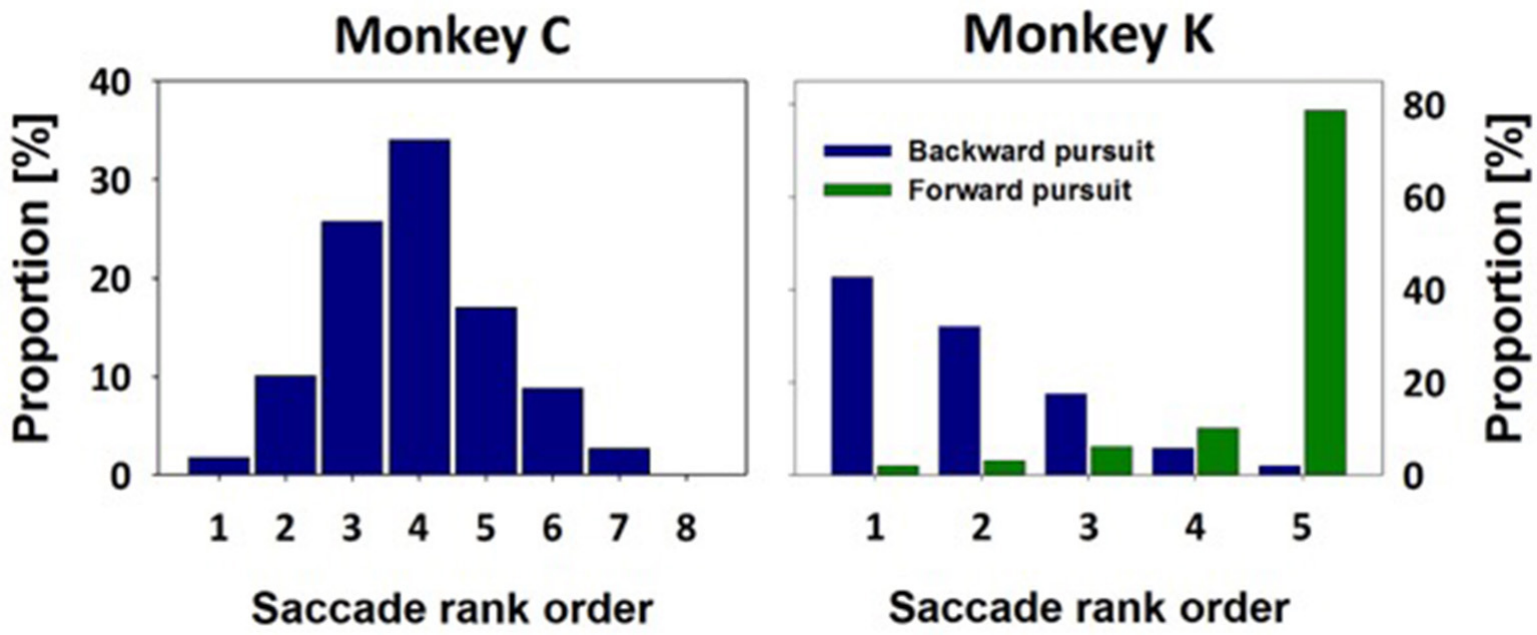
**Saccade amplitudes in moving-target trials**. Saccade amplitudes in moving-target trials covered a broad spectrum. Amplitudes were classified in bins as determined from stationary-target trials (see methods for details). For monkey C, the target always stepped toward the most eccentric target distance and immediately thereafter started to move centripetally. Accordingly, the majority of MTTs resulted in a medium sized amplitude of the initial saccade (bin #-4). For monkey K, the target always stepped toward a central position and then started to move either in the same direction, eliciting forward pursuit, or in the opposite direction, eliciting backward pursuit. Accordingly, most of the initial saccades for forward pursuit were found to have the largest amplitude (green histogram, bin #-5). On the contrary, most of the initial saccades for backward pursuit were found to have the smallest amplitude (blue histogram, bin #-1).

The spatial location of the stationary targets and of the initial appearance of the moving visual target was different for the two monkeys. For monkey C, after initial central fixation, stationary targets appeared at one of eight eccentric distances, ranging from 6° to 20° in two-degree steps. Given the variance of saccade end-points for identical target locations (van Beers, 2007; Ohl et al., 2011), we decided to bin saccade endpoints not according to their absolute values, but rather according to their relative values, i.e., from those being closest to the initial fixation location (bin #-1) to those being furthest away from initial fixation (bin #-8). Bin sizes were adjusted as to obtain an approximately equal number of trials within each bin. In the MMTs, after initial central fixation, the target always stepped to 20° eccentricity (i.e., the most eccentric stationary target location) and immediately started to move centripetally at 30°/s. As described above, a variance in saccade onset latency induces automatically a variance in saccade amplitude in order to adequately catch-up with the moving target. Across all recordings in monkey C, we collected data from 229 moving target trials. We classified each of these 229 initial saccades according to their amplitude into one of the previously specified eight bins. The observed amplitudes occupied seven out of these eight bins with the highest proportion found for a medium value (bin #-4. Figure 4A). This value will be of critical importance for our decoding approach (see below).

**Figure 5:**
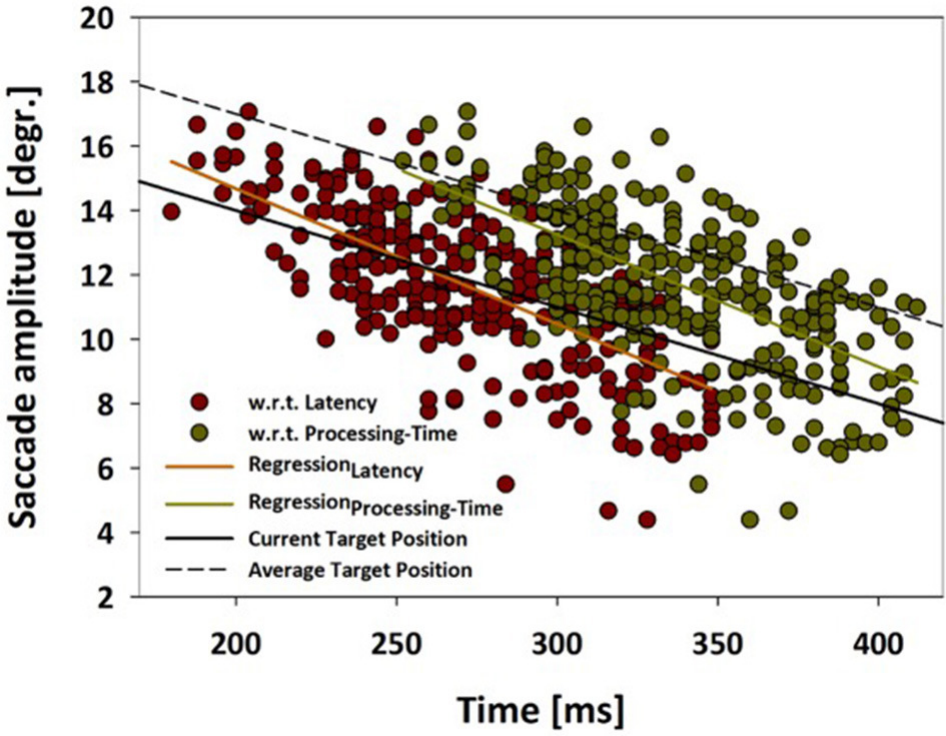
**Saccade amplitude as function of latency or processing time**. Saccade amplitudes are shown for moving target trials from monkey C as function of (i) saccade latency (red data points) or (ii) as function of processing time (green data points). Processing time is defined as the sum of the latency and the duration of an individual saccade (Quinet and Goffart, 2015). The orange (latency) and light-green (processing time) straight-lines indicate the respective linear regression functions for these two data sets. The solid black line indicates the actual target position, while the dashed black line indicates the average target position as determined from the past 200 ms. For further details, see main text.

While the above analysis determined the distribution of saccade amplitudes, it did not quantify the accuracy of the saccade landing points with respect to the moving target. Hence, in a further step, we analyzed saccade amplitudes as a function of saccade latency (dark red symbols) as well as processing time (dark green symbols) (Figure 5). This latter term has been introduced by Quinet and Goffart (2015) and is defined as the sum of the latency and the duration of an individual saccade. The orange (latency) and light-green (processing time) straight-lines indicate the respective linear regression functions for these two data sets. In the same graph, the solid black line indicates the actual target position, while the dashed black line indicates the average target position as determined from the past 200 ms. This is an average from a rather broad spectrum of values (20–500 ms) which have been suggested in the literature as an integration window for perceiving the position of (suddenly appearing) moving objects (Krekelberg and Lappe, 2001; Kerzel and Gegenfurtner, 2004). In our data set, saccade latencies varied between 180 and 348 ms, while saccade processing times ranged from 252 to 412 ms. Considering the data based on processing time (green symbols and linear regression), the amplitudes fell short off the current target position for saccades with the shortest latencies, while saccades with the longest latencies came close to the current target position. In other words: it appears as if the monkey's oculomotor system predicted the target movement, but, for the earliest saccades, could not take into account the duration of the upcoming saccade. Only for the late-onset saccades, this flight time was considered (almost) appropriately. This finding is in line with results reported by Gellman and Carl in humans (Gellman and Carl, 1991).

Due to the spatial limitation of the visual display for the measurements of monkey K, initial fixation was always eccentric. In STTs, stationary targets appeared at one of five distances (see Methods for details). Like for data from monkey C, we classified saccade amplitudes in five bins, from those being closest to the initial fixation location (bin #-1) to those, being furthest away (bin #-5). Again, bin sizes were chosen as to obtain an approximately equal number of trials in each bin. In MTTs, the target always stepped to the central of the five stationary targets and either continued to move in the direction of the step (inducing forward-pursuit) or in the opposite direction (inducing backward-pursuit). The amplitude-distributions for the initial saccades for forward- and backward-pursuit are shown in Figure 4B. Median values of the initial saccades for forward- and backward-pursuit were significantly different for each eye-movement recording as well as at the population level (*p* < 0.05 in all cases, *p* < 0.001 in most cases. Rank sum test). Importantly for our decoding approach (see below), initial saccades for forward-pursuit (*n* = 99) had in most cases an amplitude being furthest away from the fixation target (bin #-5). In contrast, initial saccades for backward-pursuit (*n* = 103) had in most cases an amplitude being closest to the fixation target (bin #-1). Again, this interim-result will be of critical importance for our decoding approach (see below).

### Decoding of target distance

Our data analysis so far had revealed that neurons in macaque area LIP show perisaccadic activity for saccades toward stationary as well as moving targets. In addition, moving target trials induced a dissociation between saccade amplitudes and the amplitude of the first appearance of the moving target. In the final step of our data analysis, we aimed to determine, whether neural discharges from a population of LIP neurons allow to decode target distance, or saccade amplitude, or both. We considered the above described activity differences in STTs and MTTs as first evidence that both values might be accessible.

**Figure 6:**
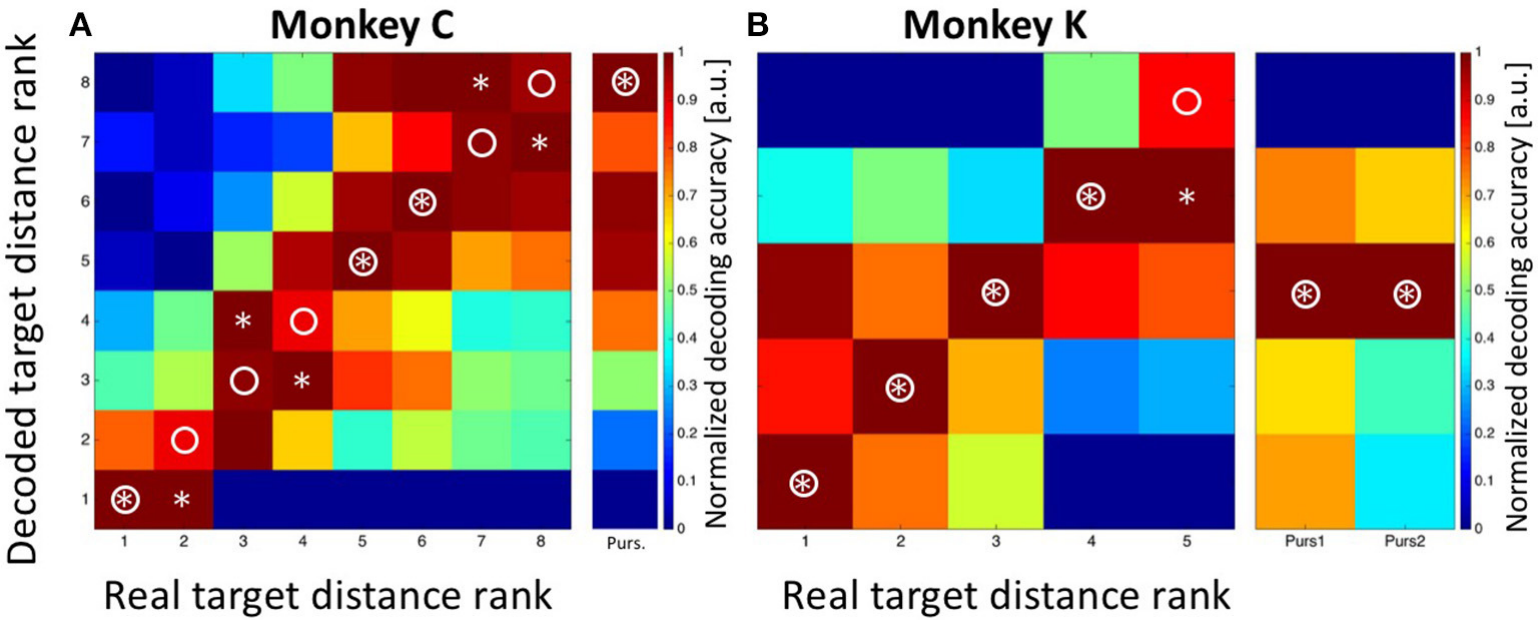
**Decoding of target distance**. Data are shown for monkey C (**A**: eight target distances) and monkey K (**B**: five target distances). The confusion matrix depicts the result from the combined Bayesian Classifier and maximum likelihood approach (see Methods for details). The values in the matrix were normalized such that for each real target distance, the decoded values ranged from 0 for the least likely decoded target distance to 1.0 for the most likely distance. Ideally, the predicted distances would always match the true target distance, and thus all observations would fall on the diagonal of the matrix (indicated by the circles). Decoded target distances are indicated by asterisks. For both monkeys, there was indeed very good correspondence between decoded and real target distances. The additional columns next to **(A,B)** indicate the result of decoding the initial target distance from the moving-target trials. These were at the most eccentric distance (bin #-8) for data from monkey C and at the middle distance (bin #-3) for data from monkey K. Importantly, decoding from moving-target trials was based on the decoder trained on stationary-target trials. As can be seen, decoded distances in moving-target trials were perfectly correct.

In a first step, we applied Bayesian statistics to all neurons with a significant tuning of their perisaccadic discharges (*n* = 33. *n*_1_ = 18 from monkey C and *n*_2_ = 15 from monkey K) in order to transform saccade-related activity into categorizations of target distances. To this end, the classifier performed a Bayes-optimal inference in which it took into account not only the likelihood of the observed population response at each of the possible target distances, but also the prior probability of each target. This prior probability function was uniform, i.e., it was 0.125 for each target for recordings in monkey C and 0.2 for each target for recordings in monkey K. The classifier used these ingredients (the likelihood function and the prior) to infer the most likely target distance given the neural data (see Methods for details). For training, we used approximately 80% of the trials recorded from each neuron. Classification was employed on the remaining 20% of the available trials from each neuron. The procedure was repeated 100 times. The results are shown in Figure 6. Classification accuracy is shown in the form of a confusion matrix, in which the distribution of predicted values (i.e., the decoded target distances) is reported for each of the real target distances. We normalized the values in the matrix such that for each real target distance, the decoded values ranged from 0 for the least likely decoded target distance to 1.0 for the most likely distance. Ideally, the decoded distances would always match the true target distance, and thus all observations would fall on the diagonal of the matrix (indicated by the circles). For both monkeys, there was indeed very good correspondence between decoded and real target distances. For monkey C, decoded values were correct in three of eight cases (Figure 6A. Decoded values are indicated by an asterisk). In the other five cases, decoded values were off by only one bin from the real values. For monkey K, decoded values perfectly matched the real values in four of five cases. In the remaining case, the decoded target distance was off by only one bin.

**Figure 7:**
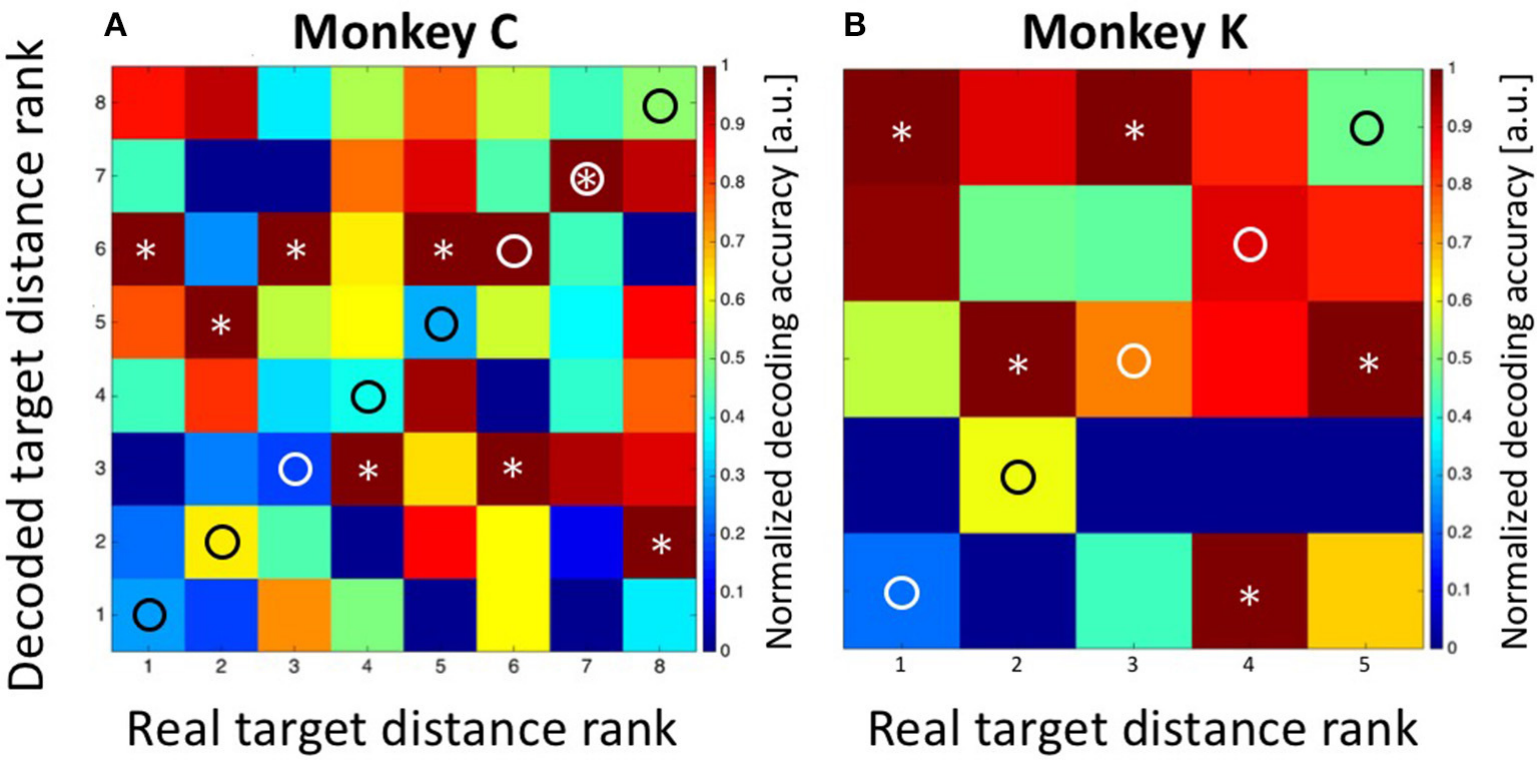
**Decoding of target distance from randomized data**. As a control concerning the performance of the decoder, we assigned the obtained discharges randomly (without replacement) to the different target distances (see Methods for details). In such case, the summed errors for data from monkey C **(A)** and monkey K **(B)** came close to the expected values in case of random performance of the decoder. True target distance ranks are marked as circles and the decoded distance ranks are marked as asterisks.

As a control concerning the accuracy of our decoding approach, we summed-up the absolute differences between the real and the decoded target distances (i.e., the bin distances) and compared this value with the one which would have been expected if the classifier had operated at chance level. For monkey C, the error was Sum_Targ.Dist.Error, Observed_ = 5^*^1 = 5 bins, which was considerably smaller than the expected error of Sum_Targ.Dist.Error, Expected_ = 21 bins in case of random performance. For monkey K, the observed sum of differences was Sum_Targ.Dist.Error, Observed_ = 1 bin, while the expected value in case of random performance would have been Sum_Targ.Dist.Error, Expected_ = 8. As a second control measure, we repeated the Bayesian computation but assigned the observed discharges in equation 2a randomly (without replacement) to any of the eight (monkey C) or five (monkey K) target distances. The result is shown in Figure 7A (data from monkey C) and Figure 7B (data from monkey K). It becomes obvious that the decoder no longer approximated the real target distances. Instead, decoding error was very large (monkey C: Sum_Targ.Dist.Error, Observed_ = 22; monkey K: Sum_Targ.Dist.Error, Observed_ = 12) and close to the expected values in case of random performance.

After having successfully decoded the distances of the stationary targets from the population activity of area LIP, we next aimed to decode the target position from the MMTs. As mentioned above, the moving target always appeared at the most eccentric bin and then moved centripetally for recordings in monkey C. For recordings in monkey K, on the other hand, the moving targets always appeared in the central amplitude bin, from where they moved either in the direction of the saccade (forward pursuit) or in the opposite direction (backward pursuit). The decoding results are visualized in the rightmost columns in Figures 6A,B. For monkey C, the maximum likelihood of the target position was found for the most eccentric target distance (bin #-8). For monkey K, the Bayesian classifier correctly decoded the starting position of the moving targets in both cases as being the central target distance (bin #-3). In other words, in all cases, the Bayesian classifier, which was trained on the data sets from the *STTs*, correctly decoded the starting position of the target from the MTTs.

### Decoding of saccade amplitude

**Figure 8:**
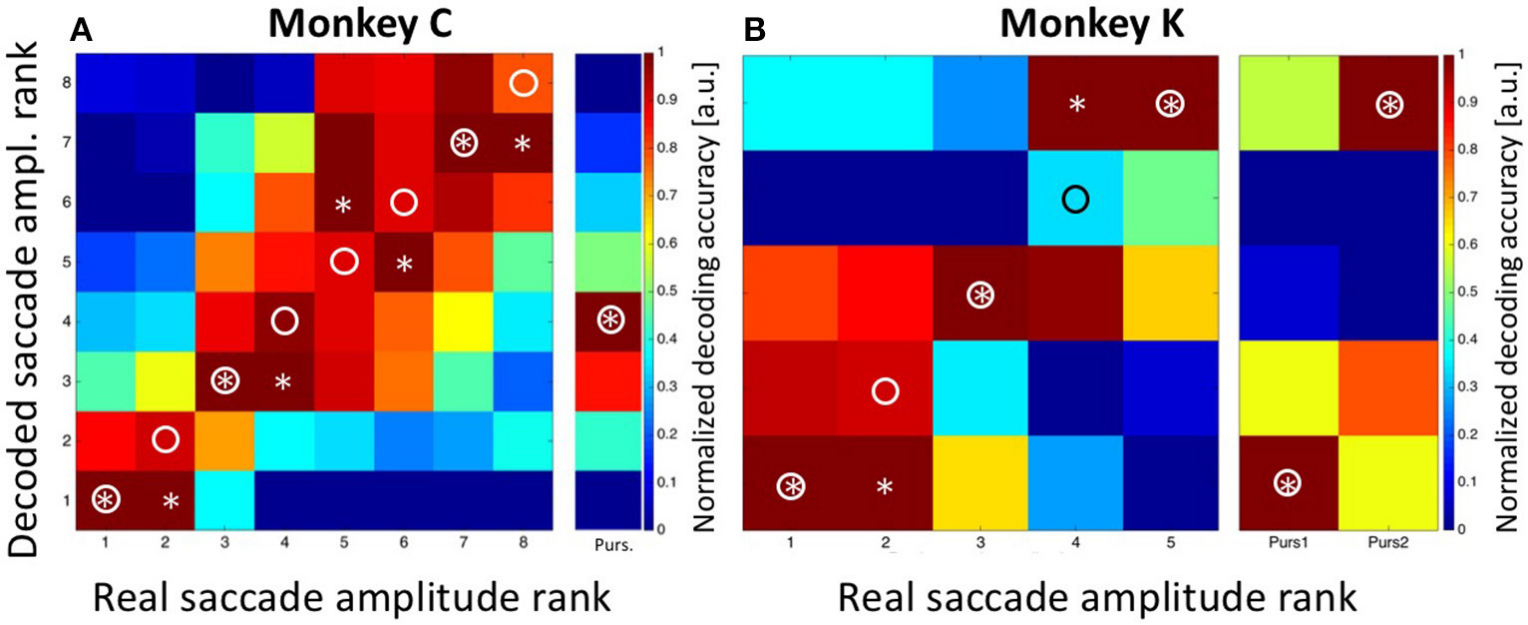
**Decoding of saccade amplitude**. Data are shown for monkey C (**A**: eight saccade amplitudes) and monkey K (**B**: five saccade amplitudes). Like in Figure 5, the confusion matrix depicts the result from the combined Bayesian Classifier and maximum likelihood approach (see Methods for details). The values in the matrix were normalized such that for each real saccade amplitude, the decoded values ranged from 0 for the least likely saccade amplitude to 1.0 for the most likely amplitude. Ideally, the predicted amplitudes would always match the true saccade amplitude, and thus all observations would fall on the diagonal of the matrix (indicated by the circles). Decoded saccade amplitudes are indicated by asterisks. For both monkeys, there was very good correspondence between decoded and real saccade amplitudes. The columns next to **A** and **B** indicate the results of decoding the amplitude of the initial saccades from the moving-target trials. For monkey C, the real value was bin #-4 (see Figure 4). For monkey K, real amplitudes were in the nearest bin (#-1) for backward pursuit and in the most eccentric bin (#-5) for forward pursuit. Importantly, decoding from moving-target trials was based on the decoder trained on stationary-target trials. In all cases, decoded saccade amplitudes in the moving-target trials were perfectly correct.

In a final step, we aimed to decode the saccade amplitude for both, the *STTs* and the MMTs. As described above, saccade amplitudes from the STTs formed a quasi-continuum. This effect is based on the fact that saccades reveal a considerable endpoint-variance (van Beers, 2007; Ohl et al., 2011). Accordingly, we determined the prior for saccade amplitude by classifying saccades as observed in the STTs either into eight bins (for monkey C) or into five bins (for monkey K), with each bin containing an approximately equal number of saccades (see Methods for details). For monkey C, saccades were correctly decoded in three of eight cases and were off by only one bin in the other five cases (Figure 8A). In Figure 8, correct saccade amplitudes are indicated by a circle, decoded amplitudes are indicated by an asterisk. summed decoding error Sum_Sacc.Amp.Error, Observed_ = 5 was noticeably smaller than the expected error Sum_Sacc.Amp.Error, Expected_ = 21 in case of random performance. Remarkably, the decoded saccade amplitude in case of MMTs was predicted to be in the fourth bin. This value is identical with the observed performance of the animal. As described above (Figure 4), saccade amplitudes toward the moving target covered a rather broad range, with the majority of trials (78/229 = 34%) being in the fourth amplitude bin.

For monkey K, saccade amplitudes for the *STTs* were correctly classified in three of five cases, and were off by only one bin for the other two cases. This error value Sum_Sacc.Amp.Error, Observed_ = 2 was considerably smaller than the expected error (Sum_Sacc.Amp.Error, Expected_ = 8) in case the classifier would have operated at random. Like for data from monkey C, saccade amplitudes were correctly classified for the MMTs, i.e., in the most eccentric bin (#-5) for forward pursuit and in the bin closest to the fovea (#-1) for backward pursuit.

**Figure 9:**
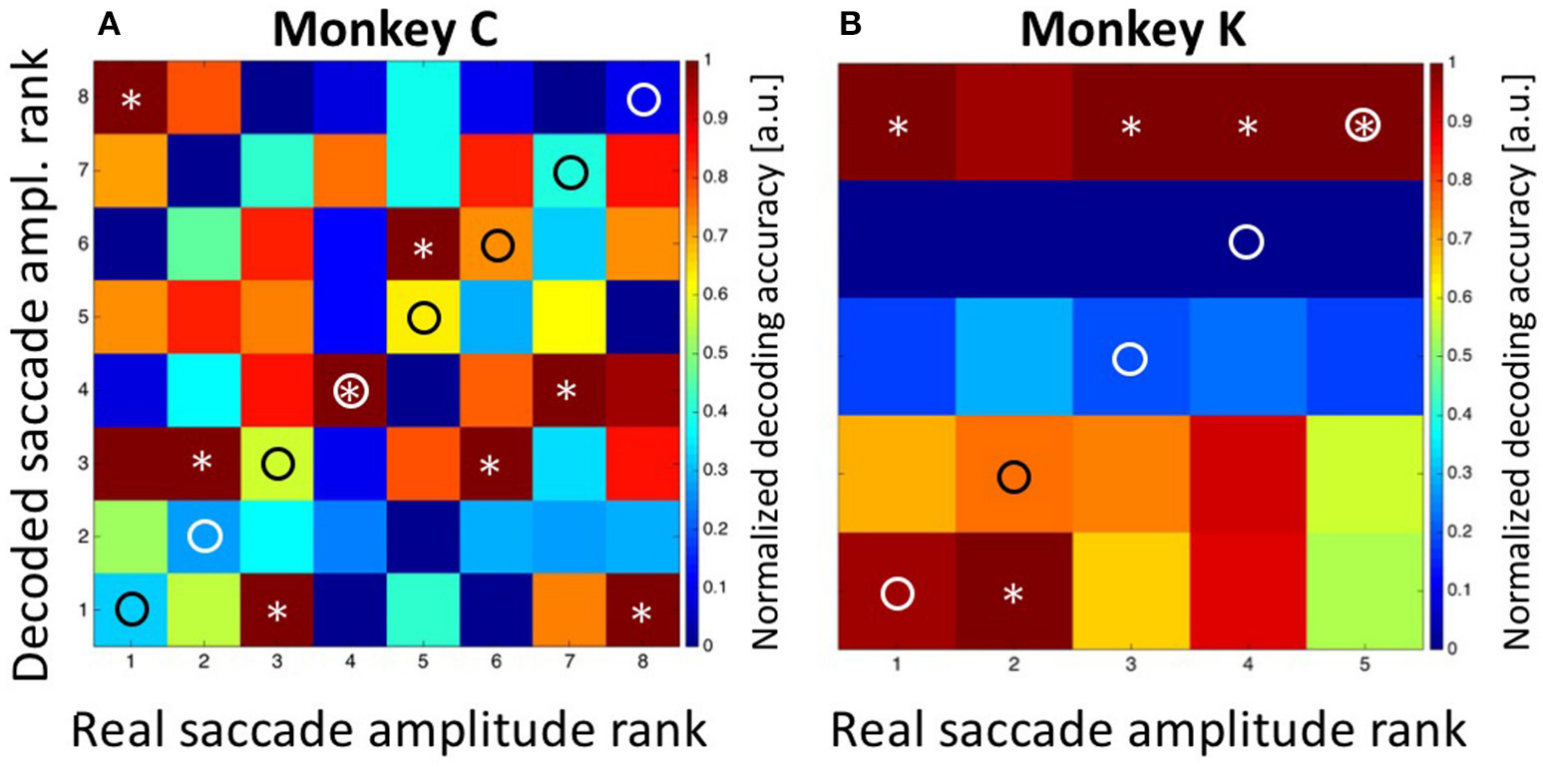
**Decoding of saccade amplitude from randomized data**. As a control concerning the performance of the decoder, we assigned the obtained discharges randomly (without replacement) to the different saccade amplitudes (see Methods for details). The summed errors for data from monkey C **(A)** and monkey K **(B)** came close to (monkey C) or were identical with (monkey K) the expected values in case of random performance of the decoder. True saccade amplitude ranks are marked as circles and the decoded amplitude ranks are marked as asterisks.

Like for target distance, we aimed for another control concerning the performance of the decoder. To this end, we randomized the assignment of observed neuronal discharges to the observed saccade amplitudes. This procedure should result in a random-like decoder performance, which we indeed observed (Figure 9). For monkey C, decoding error accumulated to Sum_Sacc.Amp.Error, Observed_ = 24, being close to the expected value of Sum_Sacc.Amp.Error, Expected_ = 21. For monkey K, decoding error accumulated to Sum_Sacc.Amp.Error, Observed_ = 8, being identical with the expected value. In summary, our data clearly show that the presaccadic neural activity from a population of LIP neurons carries simultaneously both, information about target distance and saccade amplitude.

**Figure 10:**
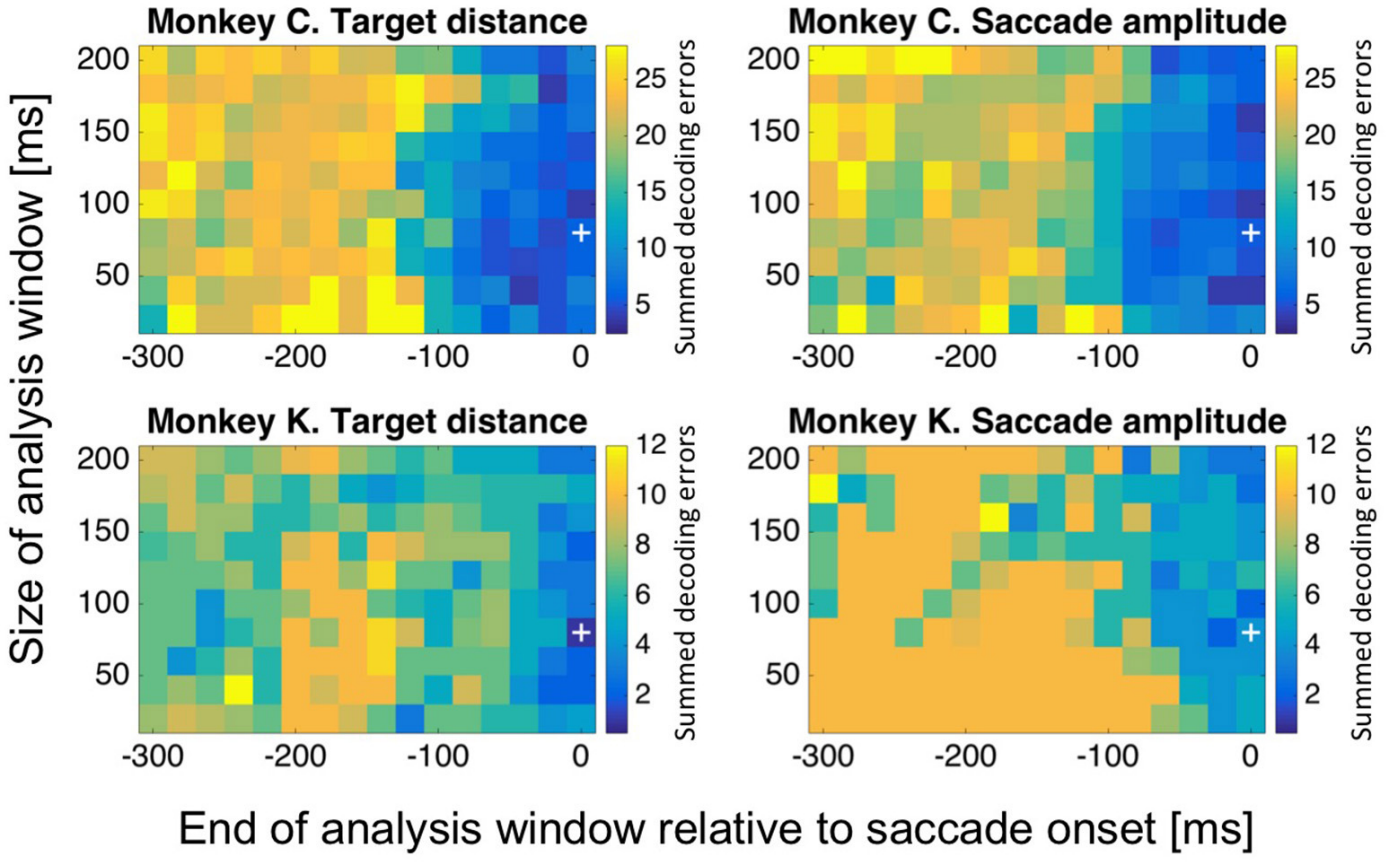
**Decoding performance as a function of the size and temporal position of the decoding window**. We aimed to determine, if decoding performance for target distance and saccade amplitude was critically dependent on the exact selection of the decoding window. To this end, we repeated our analysis for differently sized and positioned analysis windows. We varied the size of the decoding window in 20 ms steps, from 20 to 200 ms. We also shifted the end of the window from 300 ms before saccade onset to saccade onset, in 20 ms steps. For each of these 160 settings, we determined the decoding error and repeated this computation 20 times. The mean value of the summed decoding errors across all eight (monkey C) or five (monkey K) amplitude bins from these 20 repetitions for each of the 160 combinations of window size and window position relative to saccade onset are shown color-coded. For further details, see main text.

The question might arise, if decoding performance for target distance and saccade amplitude was critically dependent on the exact selection of the decoding window (−80 to 0 ms relative to saccade onset). To answer this question, we repeated our analysis for differently sized and positioned analysis windows. More specifically, we varied the size of the decoding window in 20 ms steps, from 20 to 200 ms (*n* = 10 window sizes). We also varied the temporal position of the window: we shifted the end of the window from 300 ms before saccade onset to saccade onset, in 20 ms steps (*m* = 16 temporal positions). For each of these n^*^m = 160 settings, we determined the decoding error and repeated this computation 20 times. Figure 10 shows the mean value of the summed decoding errors across all eight (monkey C) or five (monkey K) amplitude bins from these 20 repetitions for each of the 160 combinations of window size and window position relative to saccade onset. Data are shown as matrix for decoded target distance and saccade amplitude for both monkeys individually. Median onset-latency for saccades toward stationary targets was 228 ms in monkey C and 216 ms in monkey K. Accordingly, we expected that for the decoding windows ending before the average saccade latency (i.e., −220 ms and earlier), performance should be close to random. This was indeed the case for both decoded values, i.e., target distance and saccade amplitude: as detailed above, the expected sum of errors in case of random performance was *n* = 21 for monkey C and *n* = 8 for monkey K. When the analysis window was close enough to saccade onset, performance improved. The size of the analysis window had a rather marginal influence on the performance of the decoding, as performance was close to maximum even for the shortest window (20 ms).

## Discussion

We have recorded activity of neurons from macaque area LIP while animals performed saccades toward stationary and moving targets. A maximum likelihood approach revealed that presaccadic population activity in area LIP carries in parallel signals about both, target distance and the amplitude of an upcoming saccade.

### Sensory vs. motor responses

It was shown before that neurons in macaque area LIP have spatially overlapping visual receptive and saccadic motor fields (Platt and Glimcher, 1998). Accordingly, a number of studies in the past have employed sophisticated saccade paradigms (overlap saccades or memory saccades) which allow to dissociate in time visually induced from motor-related responses (e.g., Barash et al., 1991a; Colby et al., 1996). In our study, we employed exclusively visually guided saccades. This approach was on purpose. First, in our study we wanted to compare the effect of saccades toward stationary and moving targets. This approach required an immediate saccade toward a peripheral moving target, because otherwise, the animal would have had to covertly track the moving target until being allowed to approach it with a catch-up saccade. In such case, however, we would have been unable to determine the target distance as used by the animal to program its initial saccade. This, in turn, would have hindered us to answer the core question of our project, namely, if LIP population activity allows to decode target distance and saccade amplitude. Second, we considered the paradigm as employed in our study being closest to natural viewing conditions. There, an object, which enters the visual field, often triggers an immediate saccade toward it, without any externally instructed delay period. Accordingly, we decided to employ a visually guided saccade and step-ramp paradigm. We presumed that activity as recorded in our experiment, contained both, visual and motor components, which was also confirmed by our decoding analyses. As for the terminology, we decided to use the expression *saccade-related* or *peri-saccadic* activity, because activity, be it sensory or motor, was in all cases recorded along with saccades.

### Saccades toward stationary vs. moving targets

While a number of studies have investigated the directional tuning for saccades of neurons in area LIP, only few have tested the tuning for saccade amplitude (e.g., Barash et al., 1991b). In our current study, roughly one third of the neurons turned out to be tuned for saccade amplitudes between 5° and 12°. We found differential activation for saccades toward stationary and moving targets in about 40% of the cells. Activity levels were found to increase for any of the two types of saccades for approximately one half of these cells, while they decreased for the other half. As a consequence, this quasi symmetric change in responsiveness did not lead to any net difference of activity levels for the two saccade types at the population level. This result might be surprising at first glance because it suggests that information, which was available at the single cell level, seemingly disappeared at the population level. This, however, obviously was not the case because we were able to decode target distance as well as saccade amplitude from the population activity. Instead, this result is reminiscent of eye position effects which have been described also for area LIP (Andersen et al., 1990; Bremmer et al., 1997, 1998; Morris et al., 2012, 2016). These studies showed that more than 60% of the neurons in macaque area LIP carry a signal concerning the position of the eyes in the orbit. At the population level, however, this signal balances out. This is due to the fact that, roughly speaking, for any neuron revealing an increase for a certain change in eye position, there is on average another neuron with a similar decrease in activity. This causes population activity for all measured eye positions to be on average identical. Nevertheless, this LIP population activity allows decoding eye position at each instance in time and space (Morris et al., 2013). Similarly, to the eye position effects, we found roughly the same number of neurons increasing and decreasing their saccade related discharges for the two different saccade types. This population response feature allowed population decoding of target distance and saccade amplitude to be not impaired by the change of response profiles for the two saccade types at the single cell level.

The question might arise, if it is justified to employ the discharge patterns observed during saccades toward stationary targets as basis for decoding saccades toward moving targets. Differently phrased, the question is, whether neurons in area LIP change between encoding modes for different types of saccades. From our point of view, there is no ultimate answer to this question. Yet, we consider it unlikely that the encoding scheme varies dependent on the behavioral context. We have used a similar approach in a previous study dealing with the encoding of the spatial position of visual targets which were briefly flashed in the temporal vicinity of a saccade (Krekelberg et al., 2003). In this study we could show that neurons in four different extrastriate and parietal areas, among them area LIP, used identical spatial encoding regimes in different behavioral contexts, i.e., during steady fixation and perisaccadically. Likewise, we suggest that neurons employ the same code for saccades toward stationary and moving targets.

### Decoding target distance and saccade amplitude

Previous studies have aimed to determine, whether activity in area LIP encodes target distance or saccade amplitude. Results have been controversial. Platt and Glimcher (1998) argued that it is saccade amplitude which is encoded by LIP activity. The authors used a Gaussian model to fit the responses of all LIP neurons during delayed saccades to multiple targets. Similar to our current approach, they grouped neural activity by either target location or saccade amplitude. This approach, as described above, relies on the fact that saccade amplitudes for identical target distances vary on a trial by trial basis. Like in our current study, Platt, and Glimcher used this natural variability in end point scatter to distinguish the two parameters. The authors found that a Gaussian model fitted to the data accounted for slightly more response variance when based on saccade amplitude rather than on target distance.

Steenrod et al. (2013) also employed a memory saccade paradigm and found the opposite, i.e., that it is target distance but not saccade amplitude, which is encoded in macaque area LIP. Importantly, these authors separated the two parameters, i.e., target distance and saccade amplitude, by massive saccadic adaptation. In such an experimental setting, over the course of dozens of trials, the saccade target gets systematically displaced at saccade onset (for visually guided saccades) or at the end of a saccade (for memory guided saccade), thereby inducing a change in the gain of the oculomotor system. Steenrod and colleagues found that under saccadic adaptation the response profiles were unchanged as a function of target location. They concluded that neurons in LIP represent target distance, but not saccade amplitude. Importantly, both studies (Platt and Glimcher, 1998; Steenrod et al., 2013) differed from our current approach: while in the studies of Platt and Glimcher as well as Steenrod and colleagues, monkeys had to perform memory guided saccades, monkeys in our study performed visually guided saccades (for the above mentioned reasons). These memory periods in the other two studies, in principal, would have allowed for ongoing signal processing, be it e.g., a shift of an attentional spotlight or the shift of the intention to make a saccade. It is not clear from the above mentioned studies if such computations had taken place or not. In addition, in the study of Steenrod and colleagues, the employed paradigm massively changed the gain of the oculomotor system. The neural basis of these changes are as yet barely understood. They might or might not interfere with ongoing computations in area LIP. In any case: we aimed to avoid such potential interference resulting from a memory period by choosing for a visually guided saccade paradigm.

Remarkably, by employing a maximum likelihood approach, we could show that for visually guided saccades, which come close to everyday life conditions, LIP population activity carries in parallel information about both, target distance as well as the amplitude of an upcoming saccade. This result is reminiscent of a recent finding on eye position signals in monkey parietal cortex (Morris et al., 2016). Here, the authors could show that activity in four extrastriate and parietal areas of the macaque, among them area LIP, carry information not only about the current, but also about the previous and the future eye positions. In other words, eye-position information is multiplexed. Similarly, our current results show such a multiplexing of information about target distance and saccade amplitude in LIP population activity. Such multiplexing concerning visual parameters has been shown before in other visual cortical areas, among them e.g., areas V1 and MT (Zohary, 1992; Grunewald and Skoumbourdis, 2004; Smolyanskaya et al., 2013). As an example, Smolyanskaya and colleagues investigated the encoding/decoding of the direction of visual motion and binocular disparity of visual stimuli in macaque area MT. They found that the direction of motion can be read out independently from a population of MT neurons by simply averaging across the population without regard to binocular disparity and vice versa. Accordingly, the multiplexing of sensorimotor signals as shown in our study adds to previous related findings and supports the idea of multiplexing as central concept of information transfer in primate visual cortex.

### The choice of decoding parameters

After having determined the decoding performance based on the initial set of parameters, i.e., size and position of the decoding window, we aimed to find out whether our results would generalize across a larger set of parameter values, i.e., for differently sized and positioned decoding windows. We expected this to be the case based on a previous study investigating the temporal properties of visual and saccade related responses in macaque area LIP (Barash et al., 1991a). Barash and colleagues found that response onset latencies for visual stimuli from a population of LIP neurons ranged from 50 to over 200 ms. For the same neural population, the onset of saccade related discharges ranged from −200 to +200 ms relative to the onset of the eye movements. Accordingly, in a visually guided saccade paradigm with saccade latencies of about 200 ms, visual (sensory) and saccade-related (motor) responses co-exist. This result had been reason for us to assume that the discharges of the neurons of our study were multiplexed, i.e., that they contained at the same time visual and saccade related information. This assumption was confirmed by our analysis showing that target distance and the amplitude of an upcoming saccade can be decoded / predicted from the neural discharges. In such case, the exact length of the decoding window is not of critical importance.

### Directional selectivity for visual motion in area LIP

The question might arise, to what extent the directional selectivity for visual motion in macaque area LIP could have influenced our decoding results. Fanini and Assad (2009) have shown that about 60% of the neurons in area LIP are tuned to the direction of visual motion when tested with a patch of moving random dots. Different from neurons e.g., in the medio-temporal area (area MT, e.g., Albright, 1984), however, LIP neurons often reveal a substantial motion response which is independent of the direction of visual motion. Accordingly, directional selectivity in area LIP seems to be comprised of two components: a general motion component and a direction selective component. As shown by Fanini and Assad (2009), this directional component requires time to build up. In their sample, neurons achieved their maximum, steady-state directional selectivity approximately 400 ms after motion onset. In our study, median onset latencies for saccades toward moving targets were 268 ms (monkey C) or 232 ms (monkey K). We consider this first evidence that direction selective visual motion responses did not or did only marginally influence the control of the initial saccade.

Further evidence comes from the study by Quinet and Goffart (2015). These authors showed that predominantly the early visual signals of a movement trajectory are critical for guiding the initial saccade toward a moving target. Given the rather late built-up of directional selectivity of the LIP motion responses, also this result argues against an influence of the direction of visual motion of the pursuit target on the saccade related discharges in area LIP.

Along the same vein, also the visual responses employed for decoding target distance were, most likely, not or only marginally influenced by the direction of motion of the pursuit target. This view is supported by results from Kusunoki et al. (2000). These authors tested LIP neurons for directional responses with single moving stimuli (similar to the pursuit target in our study). In their analysis, Kusunoki and colleagues analyzed the first 200 ms of the motion responses. In line with the results by Fanini and Assad (2009), only marginal directional selectivity became apparent in this temporal epoch. Yet, it is roughly this very temporal interval which we employed for decoding target distance. We conclude that the selectivity for the direction of visual motion in area LIP most likely did not or did only marginally influence our decoding results.

### Saccade related activity in area LIP: an efference copy signal?

Previous studies have investigated in detail the spatio-temporal properties of saccade related discharges in monkey area LIP (e.g., Barash et al., 1991a,b). These studies revealed that neurons in area LIP, different from those in neighboring area 7a, have on average saccade related discharges starting prior to saccade onset. This might suggest that area LIP is directly involved in the neural control of saccadic eye movements. Interestingly, however, a lesion study did not find an impairment of visually guided saccades after reversible inactivation of area LIP (Wardak et al., 2002). Inactivation of macaque area LIP has behavioral consequences, though. As reported by Li and Andersen (2001), inactivation of area LIP delays the initiation of the second saccade in a double-saccade task. These functional properties, i.e., no impairment of single visually guided saccades after inactivation but impairment in a double-step saccade, are reminiscent of findings reported by Wurtz and Sommer for a thalamic structure, the medio-dorsal nucleus of the thalamus (Sommer and Wurtz, 2002, 2008; Wurtz, 2008; Cavanaugh et al., 2016). In their seminal work, and as recently summarized (Zimmermann and Bremmer, 2016), these authors have clearly worked out that neurons in the MD nucleus start to fire before the onset of saccades, while inactivation of the MD nucleus does not alter the accuracy of visually guided saccades. Sommer and Wurtz convincingly argued that the signal as provided by the MD nucleus is necessary and sufficient to keep track of the ever ongoing saccadic eye movements performed by the animal in everyday life, thereby allowing for a stable perception of the outside world. Such a signal is termed either efference copy (von Holst and Mittelstaedt, 1950) or corollary discharge (Sperry, 1950). Accordingly, the above mentioned findings imply that neural activity in area LIP carries such an efference copy or corollary discharge signal. This does not imply, however, how this functional property comes about. One obvious question would be e.g., if this property evolves in area LIP or if it results e.g., from projections from the MD nucleus of the thalamus or from the frontal eye fields. Further work is required to clarify this issue.

### An oculomotor brain machine interface

In our decoding approach, we employed presaccadic activity to decode the amplitude of an upcoming saccade. This predictive power of LIP population activity could in principal be used to guide a technical or biomedical device. A similar argument has recently been made in a study decoding saccade direction from LIP population activity (Graf and Andersen, 2014a,b). Similar to our study, these authors employed Bayesian inference from ensembles of LIP neurons to predict eye-movement plans. Graf and Andersen argued that brain-machine interfaces for eye movements might be promising aids for assisting paralyzed patients. Our current results provide further evidence for this idea. Of similar importance are findings from fMRI studies, revealing functional equivalents of macaque area LIP in human parietal cortex (Konen et al., 2004; Konen and Kastner, 2008; Sereno and Huang, 2014; Orban, 2016). One intriguing finding in this context was the ability to monitor covert intentions and, even more so, covert changes of intention based on activity in primate area LIP (Bracewell et al., 1996; Mazzoni et al., 1996; Snyder et al., 1998). In neurophysiological experiments, monkeys were instructed by appropriate cues to prepare a saccade into the preferred direction of a neuron under study or into the opposite direction. This allowed the experimenters to literally switch neural activity on and off according to the cued saccade direction. Importantly, these covert intentions were recorded during steady fixation of the animal. Similar functional properties have been reported from the functional equivalent of monkey area LIP in humans (Kleiser et al., 2009).

Different from hypothetical oculomotor brain machine interfaces (BMIs), prostheses which aim to substitute paralyzed limbs in paraplegic or tetraplegic patients have already been realized. In this field of research, two different concepts or approaches compete with each other: the one approach relies on neural activity from frontal cortex, i.e., motor or pre-motor cortex, as basis for a BMI. Already 10 years ago, Hochberg et al. (2006) have demonstrated that in a human patient with tetraplegia ensemble activity from primary motor cortex (M1) could be used to control a cursor on a computer monitor. The second approach uses recordings from parietal cortex. As an example, Aflalo et al. (2015) employed neural activity recorded from human posterior parietal cortex (PPC) to decode imagined movement trajectories. Both studies provided clear evidence that neural activity from the respective region of the brain, i.e., frontal vs. parietal cortex, can be effectively used to control a brain machine interface. From our point of view, the same conclusion most likely applies to the development of a hypothetical oculomotor BMI. While the frontal eye fields (FEF) obviously are the candidate area when deciding for frontal cortex, the parietal eye-field, i.e., area LIP, would be the candidate area when opting for parietal cortex.

Notwithstanding, more studies are required to eventually establish an oculomotor BMI. One important next step would be, e.g., to demonstrate the potential of neural discharges to allow simultaneous decoding of saccade direction and amplitude. While the results from Graf and Andersen (2014a,b) as well as our own current results are only the first steps on the long way toward such a brain machine interface, they are undoubtedly encouraging.

## Author contributions

FB designed the research. AK, SK, and JC performed the research. FB, AK, SK, and JC wrote the paper; FB contributed analytic tools and analyzed the data.

## Funding

This work was supported by Deutsche Forschungsgemeinschaft (CRC/TRR-135/A1 and RU 1847/A2).

### Conflict of interest statement

The authors declare that the research was conducted in the absence of any commercial or financial relationships that could be construed as a potential conflict of interest.
